# Treatment with cholesterol just after thawing maintains the fertility of bull sperm

**DOI:** 10.1093/molehr/gaad031

**Published:** 2023-09-01

**Authors:** Md Mazharul Islam, Takashi Umehara, Natsumi Tsujita, Masanori Koyago, Masayuki Shimada

**Affiliations:** Laboratory of Reproductive Endocrinology, Department of Bioresource Science, Graduate School of Biosphere Science, Hiroshima University, Higashi-Hiroshima, Hiroshima, Japan; Department of Animal Breeding and Genetics, Bangabandhu Sheikh Mujibur Rahman Agricultural University, Gazipur, Bangladesh; Laboratory of Reproductive Endocrinology, Department of Bioresource Science, Graduate School of Biosphere Science, Hiroshima University, Higashi-Hiroshima, Hiroshima, Japan; Graduate School of Integrated Sciences for Life, Hiroshima University, Higashi-Hiroshima, Hiroshima, Japan; Graduate School of Integrated Sciences for Life, Hiroshima University, Higashi-Hiroshima, Hiroshima, Japan; Graduate School of Integrated Sciences for Life, Hiroshima University, Higashi-Hiroshima, Hiroshima, Japan; Livestock Improvement Association of Japan Inc., Tokyo, Japan; Laboratory of Reproductive Endocrinology, Department of Bioresource Science, Graduate School of Biosphere Science, Hiroshima University, Higashi-Hiroshima, Hiroshima, Japan; Graduate School of Integrated Sciences for Life, Hiroshima University, Higashi-Hiroshima, Hiroshima, Japan

**Keywords:** cholesterol, IVF, mitochondria, sperm motility, spermatozoa

## Abstract

Freezing and thawing diminish sperm motility and fertility by disrupting the cholesterol balance in sperm plasma and organelle membranes. The aim of this study was to elucidate the mechanisms through which exogeneous cholesterol treatment enhances the quality of frozen-thawed bull sperm. The incorporation of cholesterol was investigated using boron-dipyrromethene (BODIPY)-cholesterol, and BODIPY signals were detected not only in the plasma membrane but also in the midpiece region immediately after thawing. The positive signal of cholesterol in the midpiece region was inhibited by a scavenger receptor class B Type I (SR-BI) inhibitor, block lipid transport 1 (BLT-1). To comprehend the role of exogenous cholesterol in the functions of the plasma membrane, propidium iodide (PI)/Annexin V and peanut agglutinin lectin (PNA) staining were performed. The results showed that treatment with exogenous cholesterol increased the number of acrosome-intact sperm and decreased the number of sperm with damage to the plasma membrane. Moreover, since BODIPY signals were also observed in the midpiece region, mitochondrial function was evaluated using a flux analyzer and a flow cytometer with 5,5′,6,6′-tetrachloro-1,1′,3,3′-tetraethylbenzimidazolyl carbocyanine iodide (JC-1) staining, revealing an increase in the number of sperm with high-mitochondrial activity and oxygen consumption. Finally, to assess sperm fertility, computer-assisted sperm analysis (CASA) and IVF were carried out. Sperm velocities and fertilization rates in IVF were significantly enhanced by the addition of cholesterol just after thawing. Thus, the treatment with cholesterol after thawing protected the plasma membrane from the stress of thawing and maintained mitochondrial function, thereby preserving the fertilization ability of frozen-thawed bull sperm for conventional IVF and artificial insemination (AI). Therefore, the application of cholesterol just after thawing is a promising option for improving the fertility of frozen-thawed sperm.

## Introduction

Mammalian sperm are produced in the testes; however, sperm collected from the testes cannot enter oocytes even though meiosis is completed (Brackett *et al.*, 1978a). Testicular sperm are transported to the epididymis from the testis, and the sperm gain motility and fertilization ability in the epididymis by membrane and cytoplasm remodeling (Björkgren and Sipilä, 2019). Further maturation occurs after ejaculation, and exposure to seminal plasma immediately changes the characteristics of mature sperm (Druart and de Graaf, 2018; Álvarez-Rodríguez and Martinez-Pastor, 2021). Seminal plasma is mainly produced in seminal vesicles and the prostate, and it is composed of many energy sources, such as fructose, amino acids, and fatty acids (Fu *et al.*, 2019). Additionally, a high level of cholesterol is contained in seminal plasma (Beer-Ljubić *et al.*, 2009), and the cholesterol ratio in the sperm plasma membrane is strongly associated with membrane permeability (Ehrenwald *et al.*, 1988a,b; Purdy *et al.*, 2005). Interestingly, seminal plasma suppressed the membrane permeability in mammalian, bull, boar, and ram sperm (Babcock *et al.*, 1979; Maxwell *et al.*, 1996), indicating that the permeability of the sperm membrane might be suppressed by the cholesterol in the seminal plasma. The low membrane permeability of sperm is sustained in the uterus; however, the cholesterol ratio is dramatically decreased in the oviduct (Ehrenwald *et al.*, 1990). The decline in cholesterol then transiently increases the membrane permeability and the intake of calcium ions into the sperm (Witte and Schäfer-Somi, 2007). The increased level of calcium ions strongly induces capacitation, which is characterized by hyperactivation and the acrosome reaction. Capacitation is essential for the penetration of sperm into an oocyte (Marquez and Suarez, 2004; Purdy and Graham, 2004), indicating that the sequential regulation of cholesterol in the sperm plasma membrane is essential for successful fertilization in the male/female reproductive tracts.

Cryopreservation of sperm is a useful technology not only in humans but also in livestock animals for the preservation of male fertility and effective utilization of male gametes; however, the motility and fertilization ability of cryopreserved sperm are lower than those of fresh sperm (Hammerstedt *et al.*, 1990; Medeiros *et al.*, 2002). The cholesterol level in the sperm plasma membrane is dramatically decreased during the freezing and thawing processes (Moore *et al.*, 2005; Srivastava *et al.*, 2013). During the freezing/thawing process, the formation of ice crystals causes physical disruptions in the cell membranes, leading to the release and redistribution of cholesterol molecules, and the physical stress and subsequent phase changes further facilitate the removal of cholesterol from the membranes. This may occur through the dissolution of cholesterol or its reorganization within the lipid bilayer, resulting in a decreased cholesterol content in the sperm membrane (Purdy and Graham, 2004). With the decline in cholesterol, nonphysiological capacitation, called cryo-capacitation, occurs just after thawing of frozen sperm, wherein the increase in sperm motility is transient, and most sperm lose their motility within 60 min (Srivastava *et al.*, 2013; Longobardi *et al.*, 2017). Therefore, many more sperm are required for not only *in vitro* fertilization but also successful pregnancy induction by artificial insemination (AI) when frozen-thawed sperm are used, compared with when fresh sperm are used (Vishwanath and Shannon, 2000). Thus, the decline in cholesterol in the sperm plasma membrane may be a limiting factor of fertilization ability after the freezing and thawing process. Additionally, seminal plasma improves the pregnancy ratio in porcine AI not only when epididymal sperm are used but also when frozen-thawed sperm are used (Okazaki *et al.*, 2009, 2012). After ejaculation, the membrane permeability is decreased due to the increased ratio of cholesterol (Purdy *et al.*, 2005; Srivastava *et al.*, 2013). The cholesterol ratio is lower in frozen-thawed sperm than in fresh ejaculate (Srivastava *et al.*, 2013). Therefore, the characteristics of frozen-thawed sperm are similar to those of epidydimal sperm, and the low fertilization ability of frozen-thawed sperm may be rescued by exogenous cholesterol via repair of the plasma membrane.

Research focusing on the treatment of frozen sperm with exogenous cholesterol has reported that the addition of cholesterol to the freezing extender improves sperm quality after thawing (Purdy and Graham, 2004; Rajoriya *et al.*, 2016; Yadav *et al.*, 2017). However, as cholesterol solutions are difficult to prepare, information on cholesterol inclusion during the thawing process remains scarce. In our previous study, the addition of a lipid mixture (LM) including several fatty acids, phospholipids, and cholesterol to the thawing medium increased sperm motility and survivability (Islam *et al.*, 2021). Interestingly, the improvement of sperm function by adding fatty acids was limited compared with that achieved by the LM (Islam *et al.*, 2021), suggesting that it is the exogenous cholesterol which improves sperm function just after thawing. Moreover, cholesterol is a component not only of the cellular membrane but also of the mitochondrial membrane and the secretory granule membrane. If cholesterol transporters are present in sperm, cholesterol might be incorporated into sperm and improve organelle functions. Thus, in this study, to test this hypothesis, boron-dipyrromethene (BODIPY)-cholesterol only was added to bovine sperm just after thawing, and its incorporation was analyzed. Because the incorporation of cholesterol was detected, the membrane potential and mitochondrial activity was analyzed using cholesterol lipid concentrate for cell culture, which is a useful cholesterol mixture for easy use of cholesterol. Moreover, sperm motility parameters and sperm fertilization ability were analyzed by treatment with cholesterol lipid concentrate for a short time after thawing.

## Materials and methods

### Materials

Routine chemicals were obtained from FUJIFILM Wako Pure Chemical Corporation (Osaka, Japan) and Nacalai Tesque, Inc. (Kyoto, Japan). Frozen straws of bovine sperm were kindly gifted by the Livestock Improvement Association of Japan, INC (Tokyo, Japan). Additionally, the sperm motility was assessed before every experiment, and all experiments were performed when the semen showed a motility over 30%.

### Sperm preparation and incubation

Frozen bull semen was thawed in water at 37°C for 15 s and then immediately diluted with 6 ml of first thawing medium. As the base medium, modified Bracket-Oliphant (BO) medium (Brackett *et al.*, 1978b), consisting of 112 mM NaCl, 4 mM KCl, 2.2 mM CaCl_2_, 0.8 mM NaH_2_PO_4_, 0.5 mM MgCl_2,_ 36 mM NaHCO_3_, was used. In the first thawing medium, 250× cholesterol lipid concentrate (12531018, Gibco, Grand Island, NY, USA) and/or 0.1 or 1.0 µM block lipid transport 1 (BLT1), a specific inhibitor of class B scavenger receptor type I (SR-BI), which is well known as the high-density lipoprotein receptor (SML0059, Sigma–Aldrich, St Louis, MO, USA), was added to the base medium. The first thawing medium containing frozen-thawed sperm was centrifuged at 500*g* (5 min, 37°C), and then the sperm pellet was washed twice with the base medium. After centrifugation, the sperm pellet was resuspended in the base medium, and the sperm were used for all analyses except for the quantification of the intake of cholesterol using BODIPY-cholesterol. For sperm motility analysis, sperm at 1.0 × 10^6^ sperm/ml were incubated for 120 min at 37°C under a humidified atmosphere of 5% CO_2_ in air according to our previous reports (Islam *et al.*, 2021).

### Sperm preparation and incubation of fresh sperm

Fresh bovine sperm were kindly gifted by the Livestock Improvement Association of Japan, INC (Tokyo, Japan). The semen was washed with the base medium containing 0.1% of 250× cholesterol lipid concentrate (Gibco) once, and then the sperm pellet was washed twice with the base medium. After 30, 60, and 120 min, the sperm motility was analyzed by computer-assisted sperm analysis (CASA). To assess the incorporation of cholesterol, the fresh semen was washed with the base medium containing 1 μg/ml BODIPY-cholesterol, and then analyzed by flow cytometry.

### Measurement of total cholesterol after washing with cholesterol

The concentration of total cholesterol was measured using the Total Cholesterol Assay kit (Cell Biolabs. Inc., CA, USA). Briefly, sperm at 1.0 × 10^7^ sperm/ml were washed with the base medium with or without 0.1% of 250× cholesterol lipid concentrate, and then washed twice with the base medium. After homogenization, the sample was used in the Total Cholesterol Assay kit according to the assay manufacturer’s instructions.

### Detection of sperm motility by a CASA system

Sperm motility was evaluated using CASA according to our previous study (Islam *et al.*, 2021). The sample (10 µl) was placed in a prewarmed counting chamber (depth: 20 µm) to take the CASA reading after incubation of sperm for different time intervals. Sperm tracks (0.5 s, 45 frames) were captured at 60 Hz according to our previous study using a CASA system (HT CASA-CerosII; Hamilton Thorne, Beverly, MA, USA). A minimum of three areas were captured in each sample, and more than 200 trajectories were recorded.

### Flow cytometry analysis

Flow cytometry analysis was performed using an Attune^®^ NxT Acoustic Focusing Cytometer (Invitrogen, Carlsbad, CA, USA) according to the recommendations of the International Society for Advancement of Cytometry (Lee *et al.*, 2008). Debris and agglutination were gated out using a dot graph of forward scatter (FSC) and/or side scatter (SSC). The gating strategy is shown in Supplementary Fig. S1A. The flow rate was set to 200 µl/min, and 20 000 sperm were analyzed.

#### Mitochondrial activity

Mitochondrial activity of sperm was measured using a MitoPT^®^ JC-1 Assay Kit (911, Immuno Chemistry Technologies, LLC, Bloomington, MN, USA) according to our previous study (Zhu *et al.*, 2019). Briefly, sperm at 1.0 × 10^7^ sperm/ml were incubated with 200 µl of 1 ×  working solution containing 5,5′,6,6′-tetrachloro-1,1′,3,3′-tetraethylbenzimidazolyl carbocyanine iodide (JC-1) dye at 37°C for 30 min in the dark. The sperm suspension was centrifuged (5 min, 37°C, 500*g*) and washed twice with the base medium. After washing, the sperm pellet was resuspended in the base medium and analyzed with an Attune^®^ NxT Acoustic Focusing Cytometer (Invitrogen) using a 488-nm laser and a filter with a bandwidth of 574/26 nm. The intensity of the average value was analyzed as the mean fluorescence intensity (MFI) of JC-1 orange aggregates. The sperm population is shown in Supplementary Fig. S1B.

#### Annexin V and propidium iodide staining

Sperm membrane permeability was measured using an Annexin V conjugate with an Alexa Fluor^®^ 647 Kit (A23204; Invitrogen, Molecular Probes). Briefly, sperm at 1.0 × 10^7^ sperm/ml were incubated with 1 ×  working solution containing Annexin V with Alexa Fluor^®^ 647 and propidium iodide (PI) at 37°C for 30 min in the dark. The sperm suspension was centrifuged (5 min, 37°C, 500*g*) and washed twice with the base medium. After washing, the sperm pellet was resuspended in the base medium and analyzed with an Attune^®^ NxT Acoustic Focusing Cytometer using a 488 nm laser and a filter with a bandwidth of 574/26 nm for detection of PI and a 637 nm laser and a filter with a bandwidth of 670/14 for detection of Annexin V with Alexa Fluor^®^ 647. The sperm population is shown in Supplementary Fig. S1C.

#### Quantification of the intake of cholesterol with quenching

BODIPY-cholesterol (TopFluor^®^ Cholesterol 810255, Nanocs, NY, USA) was used for the quantification of the incorporated cholesterol. BODIPY-labeled cholesterol was dissolved by dimethyl sulfoxide at 50 mg/ml, and it was used as the stock solution. Just after thawing of the frozen-thawed bovine sperm, the sperm were washed with base medium containing BODIPY-cholesterol at different concentrations. After centrifugation for 5 min (500*g*, 37°C) and washing twice with base medium to remove excess BODIPY-cholesterol, trypan blue staining (0.4%) (355-25, Nakalai Chemicals Ltd, Kyoto, Japan) was performed to quench fluorescence signals on the surface of the sperm to detect the actual region of cholesterol uptake. The fluorescence level was analyzed with an Attune^®^ NxT Acoustic Focusing Cytometer using a 488 nm laser and a filter with a bandwidth of 530/30 nm, and the intensity of the average value was measured. The sperm population is shown in Supplementary Fig. S1D. Digital images were captured using a Nikon confocal microscope after nucleic visualization by Hoechst33342 (346-0795, Dojindo, Kumamoto, Japan).

#### Quantification of intracellular calcium

Just after thawing of frozen-thawed bovine sperm, the sperm were washed with base medium with/without cholesterol lipid concentrate (250 × ). After centrifugation for 5 min (500*g*, 37°C) and washing twice with base medium, the sperm were suspended in base medium containing Fluo4-AM (F312, Dojindo) and incubated for 15 min. After centrifugation for 5 min (500*g*, 37°C) and washing twice with base medium, the fluorescence level was analyzed by an Attune^®^ NxT Acoustic Focusing Cytometer using a 488 nm laser and a filter with a bandwidth of 530/30 nm and measured as the intensity of the average value. The sperm population is shown in Supplementary Fig. S1E.

#### Peanut agglutinin lectin staining of sperm

The presence of intact acrosomes was measured using lectin from *Arachis hypogaea* (peanut) conjugated with FITC (peanut agglutinin lectin (PNA)-FITC: Sigma-Aldrich, L7381). Briefly, the sperm were fixed with 4% paraformaldehyde for 30 min and permeabilized with 0.3% (v/v) Triton X-100 with PBS (−) for 30 min at room temperature. After washing with PBS, the sperm were incubated with PNA-FITC diluted with PBS (−) at 1 in 1000 for 30 min at 37°C. The sperm suspensions were washed three times with PBS (−), and the sperm pellets were resuspended in PBS (−). The sperm were analyzed with an Attune^®^ NxT Acoustic Focusing Cytometer using a 488 nm laser and a filter with a bandwidth of 530/30 nm. The sperm population is shown in Supplementary Fig. S1F.

### Immunofluorescence of sperm

Sperm were mounted on glass slides and air-dried. The sperm were fixed with 4% paraformaldehyde for 15 min at room temperature and then permeabilized with 0.3% (v/v) Triton X-100/PBS. The sperm were probed with anti-SR-BI antibody (Cat No. NB-400-113; Novus Biologicals, Littleton, CO, USA) at a 1:100 dilution. The specificity of this antibody was confirmed by western blotting (Supplementary Fig. S2A). After washing with 0.3% (v/v) Triton X-100 in PBS (−), the antigens were visualized with Cy3-conjugated goat antirabbit IgG (1:200, Sigma-Aldrich) and DAPI (VECTESHIELD Mounting Medium with DAPI, Vector Laboratories, Newark, CA, USA). The negative control stained with only the secondary antibody is shown in Supplementary Fig. S2B. Digital images were captured using a confocal microscope (Nikon, Tokyo, Japan).

### Oxygen consumption assay

The cellular oxygen consumption rate (OCR) was monitored in real time using a Seahorse Bioscience Extracellular Flux Analyzer (XF HS Mini, Agilent, Santa Clara, CA, USA). For the flux analyzer, NaHCO_3_-Free HTF medium was made according to a previous study (Balbach *et al.*, 2020). Frozen bovine sperm were thawed with 0.1% cholesterol as described above. After washing with NaHCO_3_-Free HTF medium, the cell numbers were quantified and diluted to 3 000 000 sperm/180 µl of NaHCO_3_-Free HTF medium. Assay plates were coated with concanavalin A (0.5 mg/ml, Fujifilm Wako Chemicals, Osaka, Japan) overnight the day before the assay. Then NaHCO_3_-Free HTF medium (180 µl) containing 3 000 000 sperm was added to each well, and the plate was centrifuged at 1000*g* (1 min, room temperature) twice to fix the sperm on the well. The assay was performed in 6 min cycles of mixing (3 min) and measuring (3 min) according to the manufacturer’s recommendations. Two inhibitors were used separately in this study, namely, oligomycin (04876, Sigma-Aldrich) and FCCP (carbonyl cyanide-p-trifluoro methoxy phenylhydrazone) (C2920, Sigma-Aldrich). The final concentration was 20 µM for these inhibitors.

Analysis was conducted using a modified method based on the Mito stress test (Gu *et al.*, 2021). Oligomycin is an inhibitor of mitochondrial complex V, which consumes oxygen and generates ATP at the oxidative phosphorylation site. Therefore, by calculating the variation in OCR induced by oligomycin, the OCR contributing to ATP production can be determined (ATP-linked OCR). FCCP induces mitochondrial depolarization, and the maximal potential is measured by FCCP treatment. Based on these calculations, the OCR associated with ATP production and the maximum capacity of sperm mitochondria was determined.

### *In vitro* maturation and *in vitro* fertilization

Bovine ovaries were collected at a local slaughterhouse, and then the ovaries were transported in PBS to the laboratory at 15°C. From 3- to 5-mm diameter antral follicles, cumulus–oocyte complexes (COCs) were collected and then washed three times with TCM-199 (12340-030, Gibco Laboratories, Grand Island, NY, USA) supplemented with 0.02 AU/ml porcine follicle-stimulating hormone (Antrin R10, Kyoritsu Seiyaku, Tokyo, Japan), 5% (v/v) FBS (SH30070, HyClone Laboratories, Logan, UT, USA), and 0.2 mM sodium pyruvate (P2256, Sigma). Then 15–20 oocytes were placed in a 100 μl drop of the maturation medium, which was covered with liquid paraffin (26137-85, Nacalai) and incubated at 38.5°C, 5% CO_2_ in humidified air for 20–22 h.

After washing with the base medium with/without cholesterol or BLT-1, the sperm pellet was resuspended with IVF100 (Functional Peptides Research Institute, Yamagata, Japan). IVF100 was the modified BO medium with added 25 mM sodium pyruvate, 0.5 mM cysteine, 5 mg/ml BSA, 5 mM caffeine, and 7.5 µg/ml heparin. The COCs were transferred into microdroplets containing spermatozoa that were thawed in cholesterol-containing or control medium and then incubated for 6 h at 38.5°C under a humidified atmosphere of 5% CO_2_ in air. To assess the effects on fertilization, two groups were utilized: a control group with a normal sperm number (1 × 10^6^ sperm/ml) and a reduced sperm number group (1 × 10^5^ sperm/ml), which was deliberately made less fertile. After *in vitro* insemination, the oocytes (or presumptive zygotes) were stripped of cumulus cells by pipetting, and then placed in a culture medium of glucose-free modified synthetic oviduct fluid (SOFaa), supplemented with 2% (v/v) basal medium Eagle (BME) essential amino acids (B6766, Sigma), 1% (v/v) minimum essential medium (MEM; 11140-050, Gibco), and 5% (v/v) fetal bovine serum. Groups of 20 presumptive zygotes were incubated in a 100 μl drop at 38.5°C in 5% CO_2_, 5% O_2_, and 90% N_2_. Under our culture conditions, the parthenogenesis rate was less than 10%.

### Statistical analysis

Statistical analyses of the data were performed using GraphPad Prism 9. All experiments were performed with three or four animal replicates. Comparisons of two groups between control and cholesterol were analyzed by unpaired Student’s *t* test. Comparisons of dose-dependent studies were performed by one-way ANOVA followed by Tukey’s *post hoc* test. The normality was confirmed by the D’Agostino-Pearson test before one-way ANOVA. The comparisons of the sperm number and cholesterol were performed by two-way ANOVA followed by Bonferroni’s posttest.

## Results

### Cholesterol was incorporated into frozen-thawed bovine sperm just after thawing

**Figure 1.**
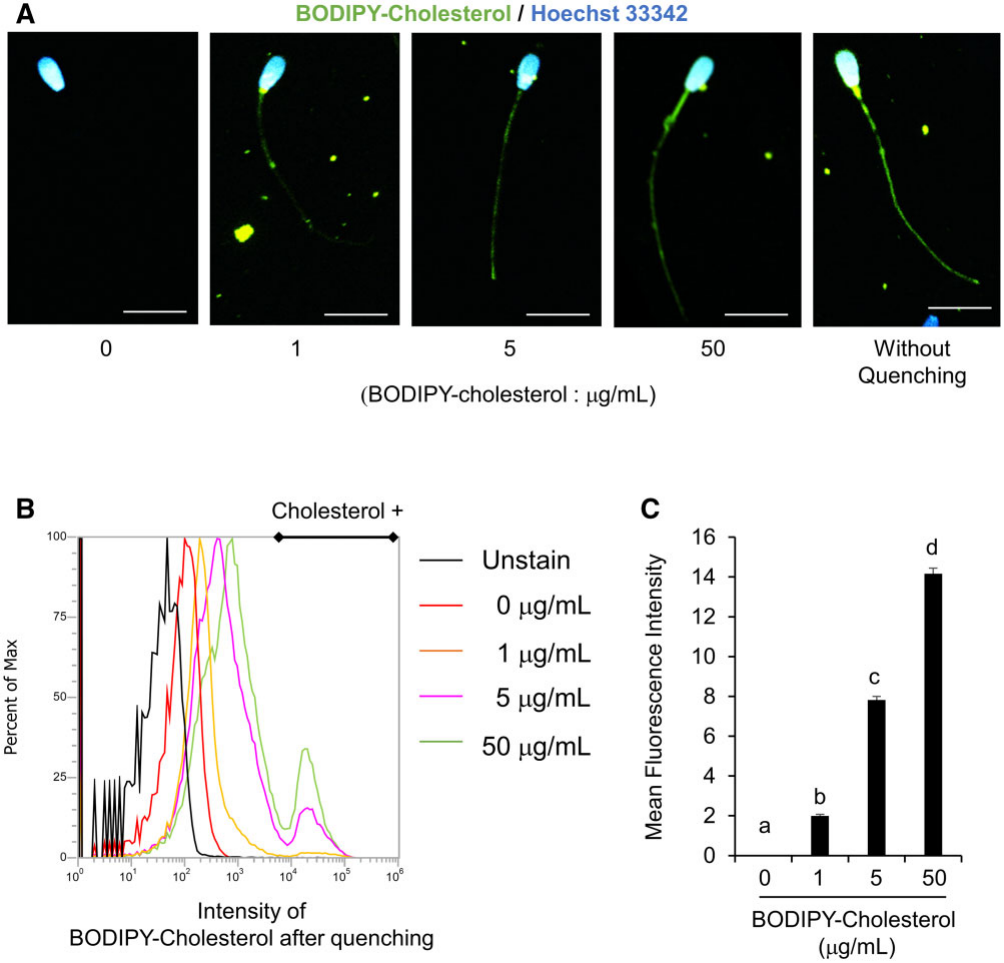
**Cholesterol was incorporated into frozen-thawed bovine sperm just after thawing process.** (**A**) Fluorescence signals with boron-dipyrromethene (BODIPY)-cholesterol at different concentrations in frozen-thawed bull sperm. Scale bar indicates 10 µm. (**B**) Overlay of fluorescence intensity peak with BODIPY-cholesterol at different concentrations after quenching. (**C**) Mean fluorescence intensity of sperm at different concentrations of BODIPY-cholesterol. Values are the mean±SEM of three animal replicates. Different superscripts denote significant differences among BODIPY-cholesterol concentrations (*P* < 0.05).

To determine the incorporation of cholesterol into frozen-thawed bovine sperm just after thawing, frozen semen was washed with medium containing BODIPY-cholesterol. BODIPY signals were observed in all regions, including the head, midpiece, and tail. After quenching, which effectively eliminated the fluorescence originating from the cell membrane surface, BODIPY signals were detected in the midpiece and tail regions of spermatozoa washed with a medium containing a concentration exceeding 5 μg/ml of BODIPY-cholesterol. Conversely, a selective presence of signals was observed solely within the midpiece regions of sperm washed with a medium containing 1 μg/ml of BODIPY-cholesterol (Fig. 1A). The positive peak of BODIPY (from 10^4^ to 10^5^) detected by flow cytometry was increased (Fig. 1B) and the mean intensities of fluorescent signals were dramatically increased in a dose-dependent manner (Fig. 1C). However, the signals of BODIPY-cholesterol into fresh sperm were found to be lower in comparison to that in frozen/thawed sperm (Supplementary Fig. S3). In order to validate the integration of cholesterol into frozen/thawed sperm, the cholesterol concentration was quantified subsequent to treatment with a 250 ×  concentration of cholesterol lipid concentrate (CLC), which resulted in a significant increase in total cholesterol levels (Supplementary Fig. S4). Therefore, exogenous cholesterol was not only bound to the surface of the cell membrane but also incorporated into the cytoplasm of frozen-thawed sperm just after thawing.

### Cholesterol protected the sperm membrane just after thawing

**Figure 2.**
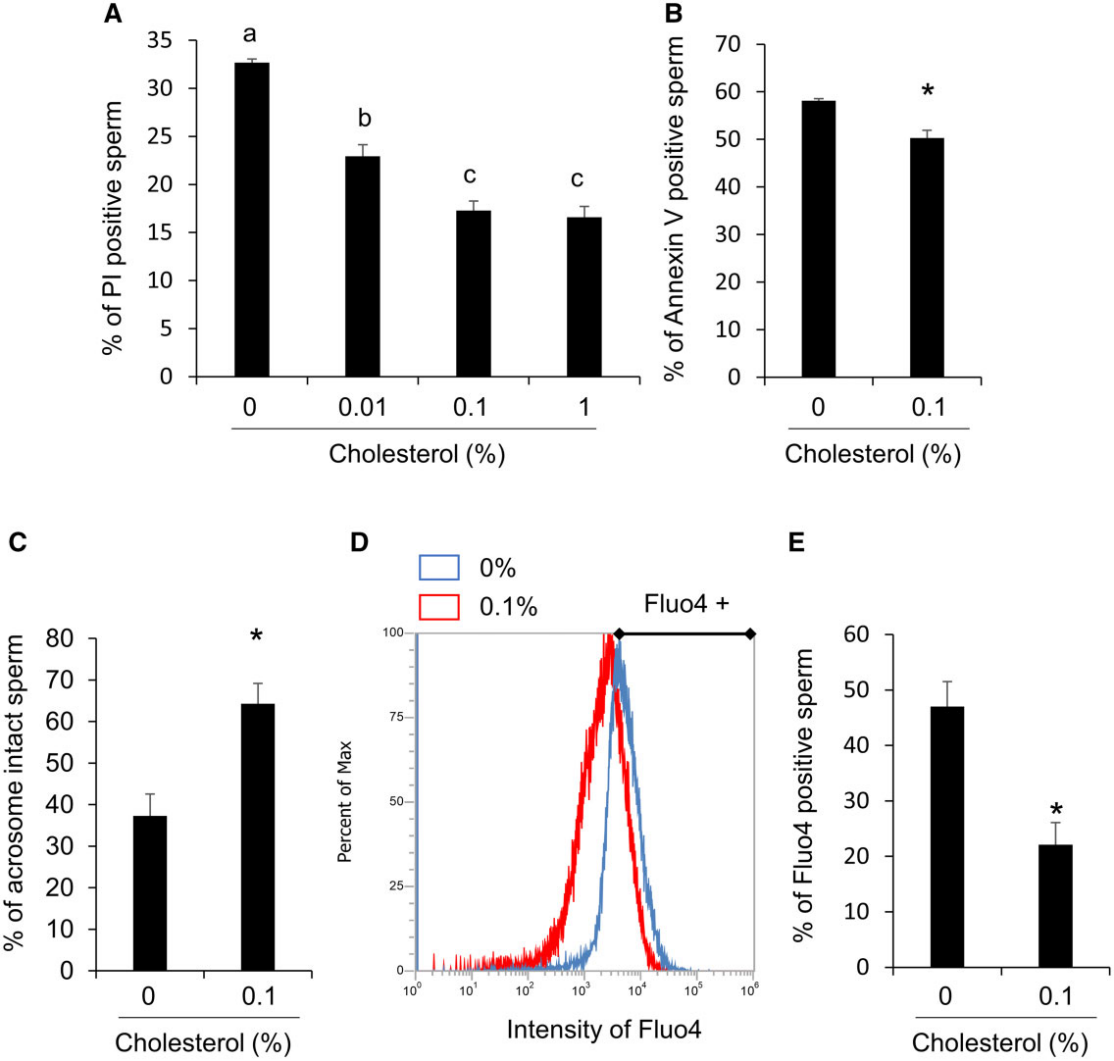
**Cholesterol protected the sperm membrane just after thawing process.** (**A**) Percent of propidium iodide (PI)-positive sperm after thawing at different concentrations of cholesterol. (**B**) Percent of Annexin V-positive sperm after thawing with/without 0.1% cholesterol. (**C**) Percent of peanut agglutinin lectin (PNA)-positive sperm after thawing with/without 0.1% cholesterol. (**D**) Overlay of fluorescence intensity peak with Fluo 4 after thawing with/without 0.1% cholesterol. (**E**) Percent of Fluo 4-positive sperm after thawing with/without 0.1% cholesterol. Values are the mean±SEM of three animal replicates. *P < 0.05 compared with 0% cholesterol.

Without cholesterol, more than 30% of sperm were PI-positive; however, the addition of cholesterol significantly decreased the ratio of PI-positive sperm (Fig. 2A). The percentage of PI-positive sperm was less than 20% after the addition of 0.1% (v/v) cholesterol (Fig. 2A). The ratio of Annexin V-positive sperm, a marker of cellular inversion, was also decreased after thawing with 0.1% (v/v) cholesterol (Fig. 2B), indicating that the addition of cholesterol just after thawing repaired the sperm plasma membrane and/or prevented further damage of the sperm plasma membrane. With the increasing quality of the sperm plasma membrane, the percentage of acrosome-intact sperm was also significantly increased with 0.1% (v/v) cholesterol (Fig. 2C). Reduced membrane integrity and acrosomal damage are strongly associated with the excessive induction of intracellular calcium (Witte and Schäfer-Somi, 2007). Thus, intracellular calcium was detected by Fluo4 staining, and the intensity was detected by flow cytometry. The results showed that the peak of Fluo4 intensity was shifted to the left by the addition of 0.1% (v/v) cholesterol to the washing medium (Fig. 2D), and the Fluo4-positive sperm ratio was significantly decreased by treatment with 0.1% (v/v) cholesterol compared with 0% (v/v) cholesterol (Fig. 2E).

### Cholesterol was incorporated into mitochondria and improved mitochondrial function

**Figure 3.**
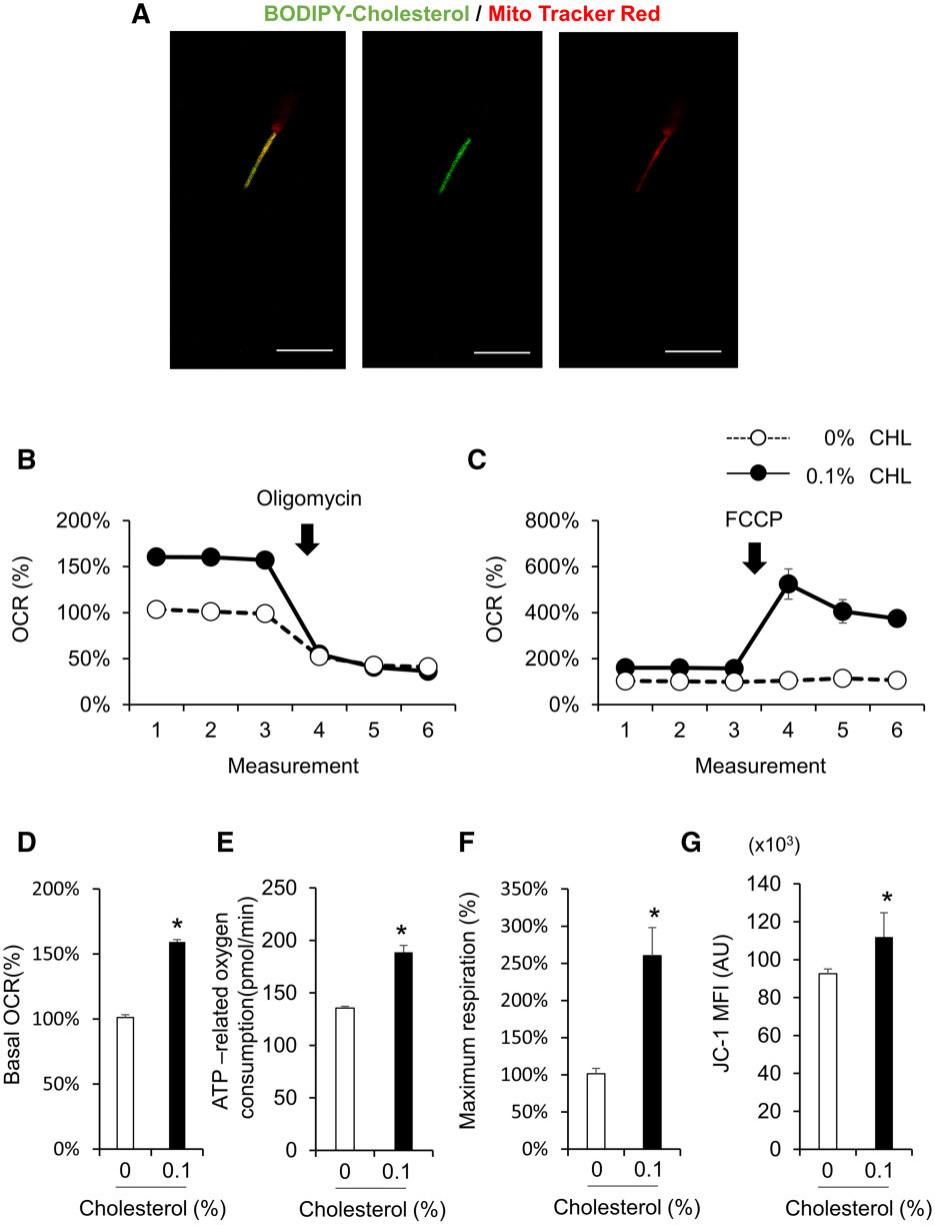
**Cholesterol was incorporated into mitochondria and improved mitochondria function.** (**A**) Co-localization of Mito Tracker Red and boron-dipyrromethene (BODIPY)-cholesterol in frozen/thawed bull sperm. Scale bar indicates 10 µm. (**B**) Tracing of oxygen consumption ratio (OCR) of frozen/thawed bull sperm washing with/without 0.1% cholesterol after oligomycin injection. (**C**) Tracing of OCR of frozen/thawed bull sperm washing with/without 0.1% cholesterol (CHL) after carbonyl cyanide p-trifluoro-methoxyphenyl hydrazone (FCCP) injection. (**D**) Basal level of OCR after washing with/without 0.1% cholesterol. (**E**) ATP production ratio calculated by the response to oligomycin after washing with/without 0.1% cholesterol. (**F**) Maximum respiration calculated by the response to FCCP after washing with/without 0.1% cholesterol. (**G**) Mean fluorescence intensity (JC-1) of mitochondrial activity after washing with/without 0.1% cholesterol. Values are the mean±SEM of three animal replicates. *P < 0.05 compared with 0% cholesterol.

Dual staining using BODIPY-cholesterol and MitoTracker Red, a marker of mitochondria, showed that BODIPY-cholesterol colocalized with mitochondria in the sperm midpiece (Fig. 3A). The OCR, which is an useful indicator of mitochondrial activity, was significantly higher in the sperm washed with 0.1% (v/v) cholesterol than in those thawed without cholesterol (Fig. 3B and C). In particular, the basal OCR ratio (the average of measurements 1, 2, and 3), ATP production ratio (the response to oligomycin, which is an inhibitor of complex V in oxidative phosphorylation (OXPHOS)), and maximum respiration (the response to FCCP) were significantly elevated in the 0.1% (v/v) cholesterol group (Fig. 3D–F). With increasing oxygen consumption upon treatment with cholesterol, the mean intensity of JC-1, a marker of activated mitochondria, was also significantly increased (Fig. 3G).

### Cholesterol was incorporated into sperm via SR-BI

**Figure 4.**
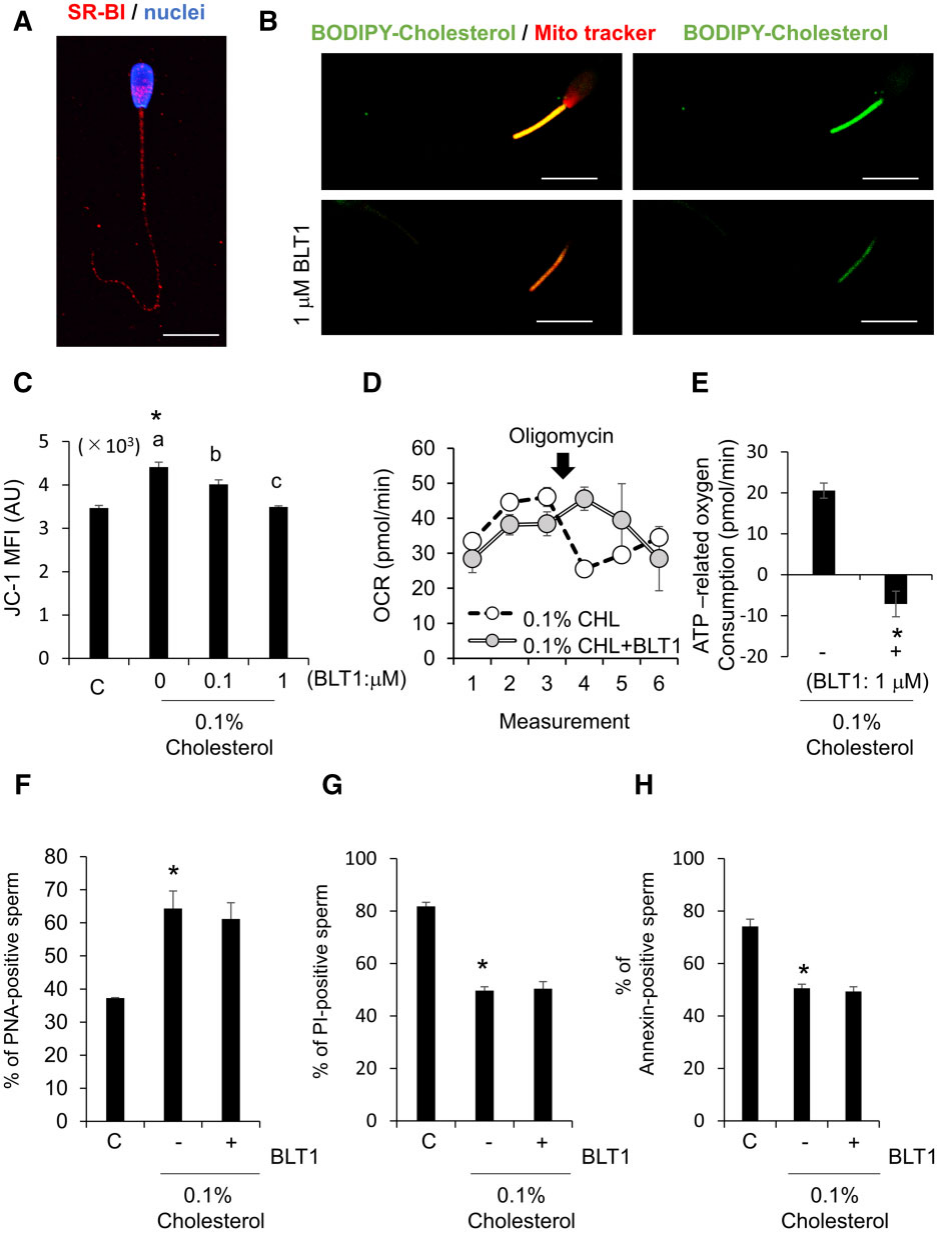
**Cholesterol was incorporated into sperm via SR-BI.** (**A**) Localization of scavenger receptor class B Type I (SR-BI) in frozen/thawed bull sperm. Scale bar indicates 10 µm. (**B**) Fluorescence of boron-dipyrromethene (BODIPY)-tagged cholesterol after washing with BODIPY-cholesterol or BODIPY-cholesterol and the inhibitor of SR-BI, block lipid transport 1 (BLT-1). (**C**) Mean fluorescence intensity (JC-1) of mitochondrial activity after washing with only cholesterol or cholesterol and BLT-1. (**D**) Tracing of oxygen consumption ratio (OCR) of frozen/thawed bull sperm washing with only cholesterol (CHL) or cholesterol and BLT-1 (CHL+BLT1) after oligomycin injection. (**E**) ATP-related OCR calculated by the response to oligomycin after washing with only cholesterol or cholesterol and BLT-1. (**F**) Percent of peanut agglutinin lectin (PNA)-positive sperm after washing with only cholesterol or cholesterol and BLT-1. (**G**) Percent of propidium iodide (PI)-positive sperm after washing with only cholesterol or cholesterol and BLT-1. (**H**) Percent of Annexin V-positive sperm after washing with only cholesterol or cholesterol and BLT-1. Values are the mean±SEM of three animal replicates. *P < 0.05 compared with C (0% cholesterol). Different superscripts denote significant differences among cholesterol concentrations (*P* < 0.05).

Immunofluorescence of SR-BI showed that SR-BI was localized in the sperm head, midpiece, and tail regions (Fig. 4A). To understand the function of SR-BI, frozen-thawed sperm were washed with both 1 μg/ml BODIPY-cholesterol and BLT1, an inhibitor of SR-BI. Upon the addition of 1 µM BLT-1, the incorporation of BODIPY-cholesterol into the sperm midpiece region was dramatically suppressed (Fig. 4B). The mean intensity of JC-1, a marker of activated mitochondria, was also significantly decreased by BLT1 in a dose-dependent manner when frozen-thawed sperm were washed with 0.1% (v/v) cholesterol (Fig. 4C). Additionally, treatment with BLT-1 eliminated the response to oligomycin in sperm washed with 0.1% (v/v) cholesterol, and the ATP production ratio was significantly decreased by BLT-1 treatment (Fig. 4D and E). Although BLT-1 treatment prevented mitochondrial recovery by washing with 0.1% (v/v) cholesterol, the ratios of PNA-positive sperm, PI-positive sperm, and Annexin V-positive sperm were not affected by BLT-1 in the presence of cholesterol (Fig. 4F–H).

### Sperm motility was increased by treatment with cholesterol just after thawing

**Figure 5.**
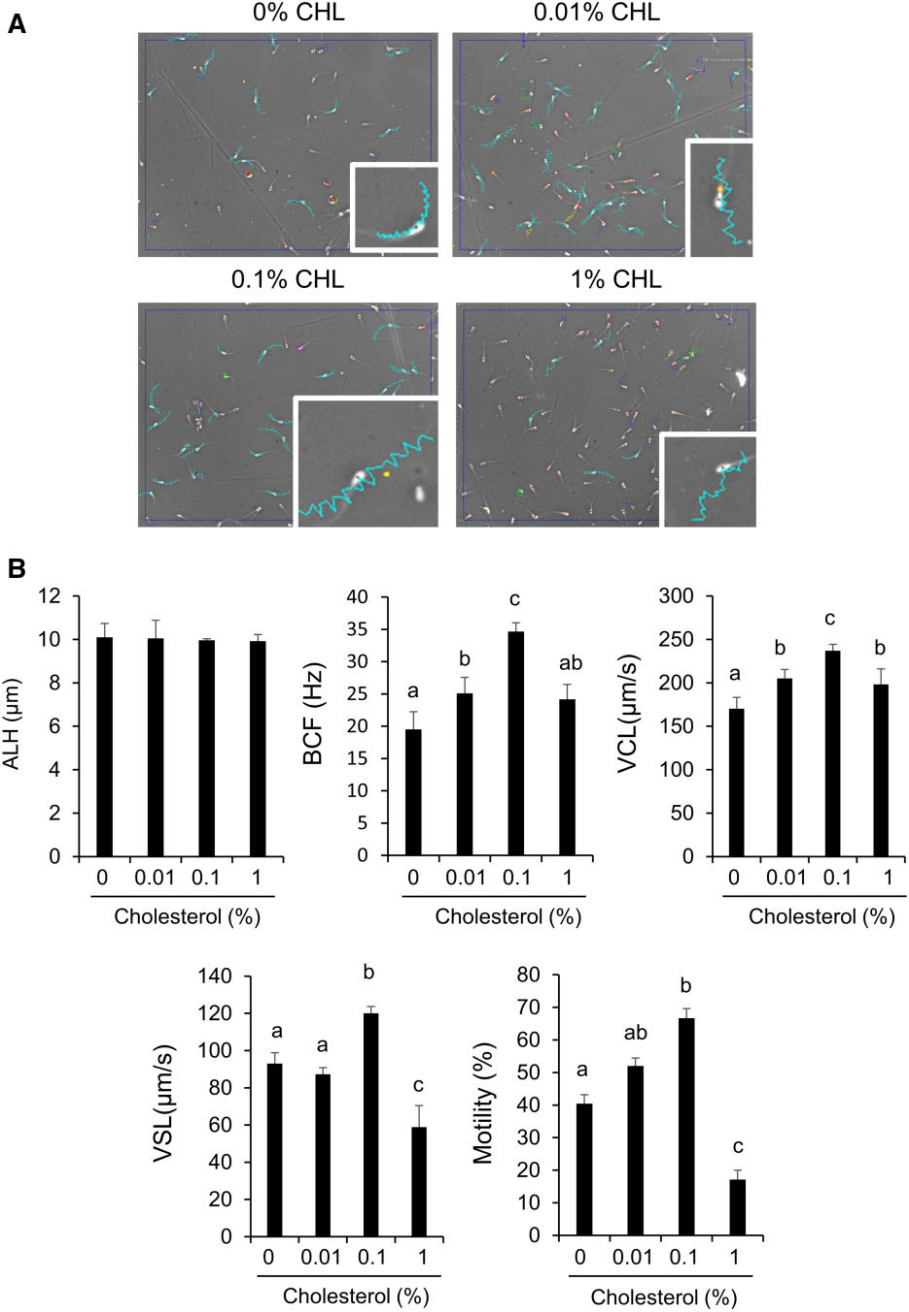
**Sperm motility was increased by the treatment with cholesterol just after thawing process.** (**A**) Sperm track after thawing at different doses of cholesterol (CHL). (**B**) Sperm parameters after thawing at different doses of cholesterol. Values are the mean±SEM of three animal replicates. Different superscripts denote significant differences among cholesterol concentrations (*P* < 0.05). ALH, amplitude lateral head displacement; BCF, beat cross frequency; VCL, curvilinear velocity; VSL, straight line velocity.

**Figure 6.**
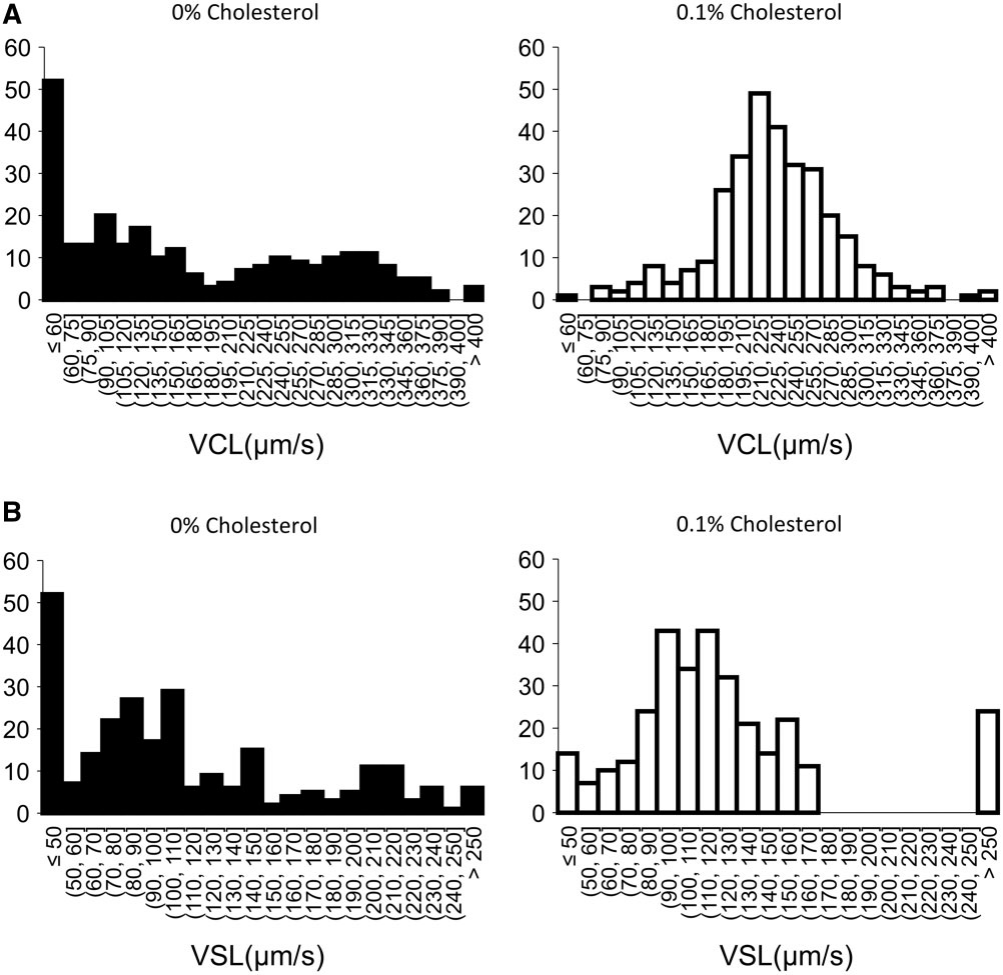
**Histograms of VCL and VSL just after washing with/without 0.1% cholesterol.**  *Y*-axis was the number of sperm, and *X*-axis was curvilinear velocity (VCL) (**A**) or straight line velocity (VSL) (**B**).

**Figure 7.**
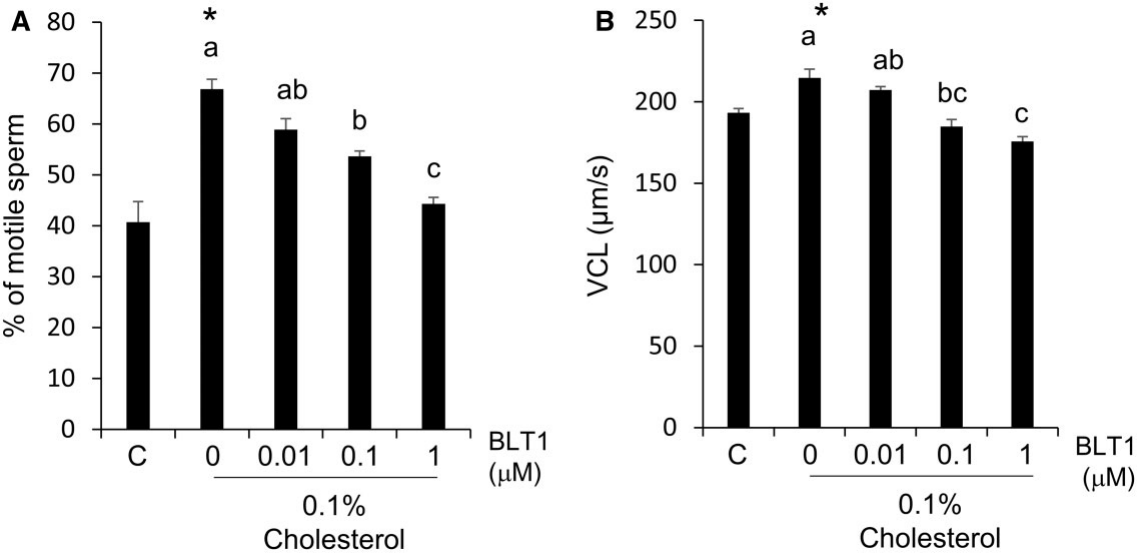
**Sperm motility after washing with cholesterol or cholesterol and BLT-1.** (**A**) Percent of motile sperm after thawing with cholesterol or cholesterol and block lipid transport 1 (BLT-1). (**B**) Curvilinear velocity (VCL) after thawing with cholesterol or cholesterol and BLT-1. Values are the mean±SEM of three animal replicates. *P < 0.05 compared with C (0% Cholesterol). Different superscripts denote significant differences among BLT-1 concentrations (*P* < 0.05).

After frozen-thawed sperm were washed with cholesterol, the sperm showed a linear pattern, and the length of the track was longer than that of the sperm thawed in 0% (v/v) cholesterol (CHL) conditions (Fig. 5A). With the addition of cholesterol to the washing medium, beat cross frequency (BCF), curvilinear velocity (VCL), and motility were significantly increased in a dose-dependent manner to 0.1% (v/v), and all parameters were decreased in the 1% (v/v) cholesterol group. VSL showed a different pattern from the other parameters; however, an increase in straight line velocity (VSL) also occurred in the 0.1% (v/v) cholesterol group (Fig. 5B). As shown in the histogram of VCL, the number of sperm that showed less than 60 µm/s was the highest in the 0% (v/v) CHL group; however, in the 0.1% (v/v) cholesterol group, the peak was observed from 210 to 225 µm/s (Fig. 6A). The histogram of VSL also showed a pattern similar to that of VCL; the number of sperm that showed less than 50 µm/s of VSL was the highest with 0% (v/v) CHL. On the other hand, the number of sperm with a VSL from 90 to 120 µm/s was the highest with 0.1% (v/v) CHL (Fig. 6B). In particular, upon washing with both cholesterol and BLT-1, the improvement of motility and VCL by cholesterol disappeared (Fig. 7A and B). After 120 min of incubation, the differences in motility parameters disappeared because the base medium did not contain any energy substrates (Supplementary Fig. S5). Additionally, the improvement of motility by exogenous cholesterol was not observed in fresh sperm (Supplementary Fig. S6).

### Cholesterol improved the fertilization ability of frozen-thawed sperm in bovine IVF

**Figure 8.**
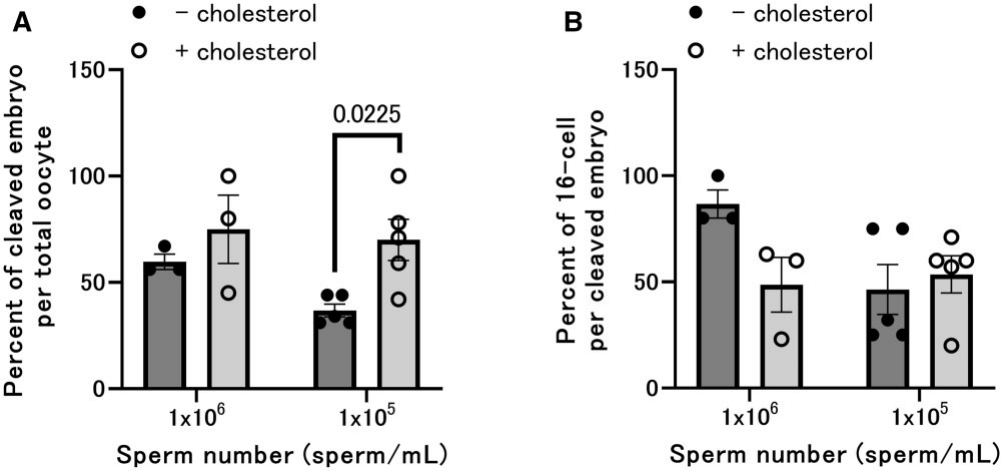
**Cholesterol improved the fertilization ability of frozen-thawed sperm in bovine IVF.** (**A**) Cleaved (2-cell) bovine embryo rate after IVF using frozen bull semen thawing with/without cholesterol. (**B**) Rate of developmental rate to the 16-cell stage per two-cell bovine embryo after IVF using frozen bull semen thawing with/without cholesterol. Values are the mean±SEM of five animal replicates.

Frozen-thawed semen was washed with 0.1% (v/v) cholesterol-containing medium and washed twice with medium without cholesterol. After centrifugation (see Materials and methods), the sperm pellet was suspended in the fertilization medium, and the sperm in the fertilization medium were used for conventional *in vitro* fertilization. The fertilization ratio was not different when 1.0 × 10^6^ sperm/ml were used between the cholesterol-free group (−) and the cholesterol-containing group (+). However, considering that the condition of 1.0 × 10^6^ sperm/ml is sufficient for successful fertilization even with normal frozen sperm, we also conducted *in vitro* fertilization with a reduced sperm count. The fertilization ratio was less than 40% when 1.0 × 10^5^ sperm/ml was used in the cholesterol-free group (−); however, the fertilization ratio was around 70% in the cholesterol-containing group (+) (Fig. 8A, Table 1). Additionally, polyspermy was not increased under cholesterol conditions (Supplementary Fig. S7B). The developmental rate to the 16-cell stage was not changed by cholesterol treatment (Fig. 8B).

**Table 1:** Effect of washing with cholesterol on IVF using different sperm numbers.

Sperm no./ml	Treatment	Total	Cleaved	Cleaved/total (%)	16-cell	16-cell/cleaved (%)
1 × 10^5^	CTRL	66	24	(36.8±2.9)	12	(46.4±11.7)
	CHL	78	51	(70.0±9.7)	29	(53.6±8.7)
1 × 10^6^	CTRL	32	18	(59.7±3.7)	15	(86.6±6.7)
	CHL	51	39	(75.0±16.1)	17	(48.7±12.9)

IVF was performed using frozen/thawed bull sperm washing with 0.1% cholesterol (CHL) or without cholesterol (CTRL).

## Discussion

During the freezing and thawing process of cells, the physicality of the membrane is shifted from the fluid phase to the gel phase by freezing-induced dehydration, and the permeability and the integrity of the plasma membrane are dramatically changed (Sieme *et al.*, 2015). This alteration is called the lipid-phase transition, which decreases the motility and fertilization ability of frozen-thawed sperm (Watson and Morris, 1987; Drobnis *et al.*, 1993). It is well known that cholesterol plays an essential role in regulating the physical condition of the cellular membrane by controlling the organization of lipids (Veatch and Keller, 2002). Interestingly, the cholesterol level in the sperm plasma membrane is also dramatically decreased by the freezing and thawing process (Srivastava *et al.*, 2013), indicating that a decline in cholesterol in the sperm plasma membrane would be a major cause of damage in frozen-thawed sperm via the lipid-phase transition.

In this study, to clarify the impact of replenishing cholesterol in the sperm cell membrane immediately after thawing on sperm functions, two different types of exogenous cholesterol were added to only the first washing medium. BODIPY-cholesterol was incorporated into both the sperm plasma membrane and the cytoplasm in the sperm midpiece region, and both the membrane quality and the mitochondrial activity were improved by washing with 0.1% (v/v) 250 ×  cholesterol lipid concentrate (CLC). Moreover, the fertilization rate after IVF using a small number of sperm (1.0 × 10^5^ sperm/ml) was significantly higher after washing with 0.1% CLC than after washing without cholesterol. Lu et al. (2011) reported that about 7 mM of cholesterol was contained in CLC, indicating that 2.7 µg/ml of cholesterol was contained in the washing medium with 0.1% of CLC (Lu *et al.*, 2011). On the other hand, in the BODIPY-cholesterol assay, the incorporation of cholesterol was observed when adding more than 1 µg/ml of BODIPY-cholesterol. Although the incorporation of BODIPY-cholesterol was increased in a dose-dependent manner up to 50 µg/ml, the positive effects disappeared with 1% CLC (27 µg/ml of cholesterol). In 1 µg/ml of BODIPY-cholesterol, 0.64 µg/ml of cholesterol was present, indicating that the appropriate concentration of exogenous cholesterol in the washing medium to sustain the fertility of frozen/thawed bull sperm was 2.7 µg/ml. Therefore, the quality and fertility of frozen-thawed sperm can be enhanced by brief exposure to 2.7 µg/ml cholesterol just after thawing. Although several studies have demonstrated that cholesterol treatment during the freezing process improves sperm membrane integrity and sperm motility, the fertilization ratio was not examined in those studies (Purdy and Graham, 2004; Moore *et al.*, 2005). Hence, this study is the first investigation to demonstrate that exogenous cholesterol just after thawing has the potential to maintain the fertility of frozen-thawed bull sperm at the appropriate concentration.

SR-BI facilitates the uptake of cholesterol from high-density lipoprotein (HDL) binding cholesterol and is therefore referred to as the HDL receptor (Ji et al., 1997). In bovine seminal plasma, approximately half of the cholesterol exists in the form of HDL-cholesterol (Beer-Ljubić *et al.*, 2009), and the concentration of cholesterol esters in spermatozoa is higher after ejaculation compared to before (Quinn and White, 1967). Additionally, our result showed that incorporation of cholesterol was inhibited by the treatment of the inhibitor of SR-BI, and this was not observed in ejaculated fresh sperm (Supplementary Fig. S3), indicating that the sperm which have low cholesterol could uptake the cholesterol via SR-BI. On the other hand, HDL is well-recognized as a regulator of cholesterol efflux (Krieger, 1999). In the context of spermatozoa, the addition of HDL during IVF has been shown to enhance cholesterol efflux and induce capacitation (Thérien *et al.*, 1997; Lane *et al.*, 1999). Although the precise interplays between the diverse functions of HDL remains unclear, our findings indicate that HDL-cholesterol present in seminal plasma might be incorporated into bovine spermatozoa via SR-BI.

Dual staining using BODIPY-cholesterol and MitoTracker Red showed that exogenous cholesterol was incorporated into not only the sperm plasma membrane but also the midpiece region's localized mitochondria via SR-BI. It is well known that cholesterol is not a major component of the mitochondrial membrane (Ardail *et al.*, 1990); however, cholesterol in the mitochondria is strongly associated with the regulation of proton leakage, which is essential for ATP production of complex V in OXPHOS (Baggetto *et al.*, 1992). Khalil *et al.* (2018) observed distorted cristae of mitochondria in frozen-thawed bull sperm. With the structural alteration of cristae, mitochondrial activities are dramatically decreased in frozen-thawed bull sperm (Khalil *et al.*, 2018). In this study, although the structure of mitochondria was not unclear, the mitochondrial membrane potential and the oxygen consumption rate were significantly increased in frozen-thawed sperm by the treatment with cholesterol, indicating that treatment with cholesterol just after thawing might improve mitochondrial function via recovery of the mitochondrial structure in the mitochondrial membrane.

During the freezing and thawing process, both mitochondria and the acrosome region are damaged (Srivastava *et al.*, 2013). Acrosomes play an important role in the fertilization process because several enzymes that are essential for the penetration of sperm into oocytes are contained in the acrosomal region (Abou-Haila and Tulsiani, 2000), suggesting that damage to the acrosome by the freezing and thawing process is one of the reasons why frozen-thawed sperm exhibit decreased fertilization ability. Acrosomes are thought to be organelles similar to lysosomes, and lysosomes are also surrounded by membranes composed of lipids and cholesterol, similar to mitochondria (Yang *et al.*, 1998; Moreno and Alvarado, 2006). In this study, we found that washing with cholesterol suppressed the damage to the sperm acrosome region, indicating that exogenous cholesterol would reinforce the lysosomal membrane in a manner similar to that for the mitochondrial membrane. Additionally, both the cleavage of the lysosomal membrane and the release of acrosomal enzymes are dependent on calcium (Jaiswal *et al.*, 2002), and the addition of calcium chelator during the thawing process suppresses the irregular acrosomal reaction (Okazaki *et al.*, 2011). We found that washing with cholesterol suppressed the elevations in sperm calcium levels, suggesting that the incorporation of cholesterol suppresses acrosomal damage by both reinforcing the acrosomal membrane and suppressing intracellular calcium. Therefore, cholesterol, just after the thawing process, plays an important role in protecting the cellular membrane and the organelle membranes, which maintains the high fertilization ability of frozen-thawed sperm.

The cholesterol ratio is also low in epididymal sperm compared with that in ejaculates (Quinn and White, 1967). The increase in cholesterol in the sperm membrane is related to exposure to seminal plasma containing more than 2 mM cholesterol (Beer-Ljubić *et al.*, 2009). Seminal plasma is important for inhibiting abnormal capacitation characteristics of sperm in the uterus because capacitated sperm enter the uterine gland and are removed by leucocytes (Akthar *et al.*, 2020). Although species differ in regard to seminal plasma entering the uterus with sperm, a role of seminal plasma in anticapacitation is common in mammals (Thérien *et al.*, 1997; Manjunath and Thérien, 2002). Interestingly, our previous study showed that the insemination of frozen-thawed boar sperm with seminal plasma dramatically improved the pregnancy ratio in AI (Okazaki *et al.*, 2012). In this study, exposure to cholesterol just after the thawing process suppressed the capacitated characteristics, similar to the effects of seminal plasma, indicating that cholesterol is an important factor in seminal plasma for transporting the sperm into the oviduct during the physiological fertilization process.

In conclusion, exogenous cholesterol delivered just after the thawing process was incorporated not only into the sperm plasma membrane but also into the midpiece region. Cholesterol improved cellular membrane integrity and increased sperm motility by upregulating mitochondrial functions via SR-BI. These positive effects of cholesterol could enable successful fertilization in IVF using a minimum number of sperm. Therefore, our investigations contribute to the development of a washing method for frozen-thawed sperm for *in vitro* fertilization and AI as well as for other types of mammalian cells with high-quality mitochondria.

## Supplementary Material

gaad031_Supplementary_Data

## Data Availability

All data needed to evaluate the conclusions in the article are present in the article and/or the Supplementary Material.

## References

[gaad031-B1] Abou-Haila A , Tulsiani DR. Mammalian sperm acrosome: formation, contents, and function. Arch Biochem Biophys 2000;379:173–182. 10898932 10.1006/abbi.2000.1880

[gaad031-B2] Akthar I , Suarez SS, Morillo VA, Sasaki M, Ezz MA, Takahashi K-I, Shimada M, Marey MA, Miyamoto A. Sperm enter glands of preovulatory bovine endometrial explants and initiate inflammation. Reproduction 2020;159:181–192. 31794421 10.1530/REP-19-0414

[gaad031-B3] Álvarez-Rodríguez M , Martinez-Pastor F. Molecular determinants of seminal plasma on sperm biology and fertility. Int J Mol Sci 2021;22:3555. 33808064 10.3390/ijms22073555 PMC8037708

[gaad031-B4] Ardail D , Privat JP, Egret-Charlier M, Levrat C, Lerme F, Louisot P. Mitochondrial contact sites. Lipid composition and dynamics. J Biol Chem 1990;265:18797–18802. 2172233

[gaad031-B5] Babcock DF , Singh JP, Lardy HA. Alteration of membrane permeability to calcium ions during maturation of bovine spermatozoa. Dev Biol 1979;69:85–93. 376375 10.1016/0012-1606(79)90276-8

[gaad031-B6] Baggetto LG , Clottes E, Vial C. Low mitochondrial proton leak due to high membrane cholesterol content and cytosolic creatine kinase as two features of the deviant bioenergetics of Ehrlich and AS30-D tumor cells. Cancer Res 1992;52:4935–4941. 1516050

[gaad031-B7] Balbach M , Buck J, Levin LR. Using an extracellular flux analyzer to measure changes in glycolysis and oxidative phosphorylation during mouse sperm capacitation. J Vis Exp 2020;**155**:e60815. 10.3791/60815 32065141

[gaad031-B8] Beer-Ljubić B , Aladrović J, Marenjak TS, Laskaj R, Majić-Balić I, Milinković-Tur S. Cholesterol concentration in seminal plasma as a predictive tool for quality semen evaluation. Theriogenology 2009;72:1132–1140. 19767087 10.1016/j.theriogenology.2009.07.009

[gaad031-B9] Björkgren I , Sipilä P. The impact of epididymal proteins on sperm function. Reproduction 2019;158:R155–R167. 31176304 10.1530/REP-18-0589

[gaad031-B10] Brackett BG , Hall JL, Oh Y-K. In vitro fertilizing ability of testicular, epididymal, and ejaculated rabbit spermatozoa. Fertil Steril 1978a;29:571–582. 668937

[gaad031-B11] Brackett BG , Oh YK, Evans JF, Donawick WJ. In vitro fertilization of cow ova. Theriogenology 1978b;9:89. 627309 10.1016/0093-691x(78)90057-2

[gaad031-B12] Drobnis EZ , Crowe LM, Berger T, Anchordoguy TJ, Overstreet JW, Crowe JH. Cold shock damage is due to lipid phase transitions in cell membranes: a demonstration using sperm as a model. J Exp Zool 1993;265:432–437. 8463792 10.1002/jez.1402650413

[gaad031-B13] Druart X , de Graaf S. Seminal plasma proteomes and sperm fertility. Anim Reprod Sci 2018;194:33–40. 29657075 10.1016/j.anireprosci.2018.04.061

[gaad031-B14] Ehrenwald E , Foote RH, Parks JE. Bovine oviductal fluid components and their potential role in sperm cholesterol efflux. Mol Reprod Dev 1990;25:195–204. 2310569 10.1002/mrd.1080250213

[gaad031-B15] Ehrenwald E , Parks JE, Foote RH. Cholesterol efflux from bovine sperm. I. Induction of the acrosome reaction with lysophosphatidylcholine after reducing sperm cholesterol. Gamete Res 1988a;20:145–157. 3235032 10.1002/mrd.1120200205

[gaad031-B16] Ehrenwald E , Parks JE, Foote RH. Cholesterol efflux from bovine sperm: II. Effect of reducing sperm cholesterol on penetration of zona-free hamster and in vitro matured bovine ova. Gamete Res 1988b;20:413–420. 3235049 10.1002/mrd.1120200403

[gaad031-B17] Fu Q , Pan L, Huang D, Wang Z, Hou Z, Zhang M. Proteomic profiles of buffalo spermatozoa and seminal plasma. Theriogenology 2019;134:74–82. 31146187 10.1016/j.theriogenology.2019.05.013

[gaad031-B18] Gu X , Ma Y, Liu Y, Wan Q. Measurement of mitochondrial respiration in adherent cells by Seahorse XF96 Cell Mito Stress Test. STAR Protoc 2021;2:100245. 33458707 10.1016/j.xpro.2020.100245 PMC7797920

[gaad031-B19] Hammerstedt RH , Graham JK, Nolan JP. Cryopreservation of mammalian sperm: what we ask them to survive. J Androl 1990;11:73–88. 2179184

[gaad031-B20] Islam MM , Umehara T, Tsujita N, Shimada M. Saturated fatty acids accelerate linear motility through mitochondrial ATP production in bull sperm. Reprod Med Biol 2021;20:289–298. 34262396 10.1002/rmb2.12381 PMC8254171

[gaad031-B21] Jaiswal JK , Andrews NW, Simon SM. Membrane proximal lysosomes are the major vesicles responsible for calcium-dependent exocytosis in nonsecretory cells. J Cell Biol 2002;159:625–635. 12438417 10.1083/jcb.200208154 PMC2173094

[gaad031-B22] Ji Y , Jian B, Wang N, Sun Y, Moya ML, Phillips MC, Rothblat GH, Swaney JB, Tall AR. Scavenger receptor BI promotes high density lipoprotein-mediated cellular cholesterol efflux. J Biol Chem 1997;272:20982–20985. 9261096 10.1074/jbc.272.34.20982

[gaad031-B23] Khalil WA , El-Harairy MA, Zeidan AEB, Hassan MAE, Mohey-Elsaeed O. Evaluation of bull spermatozoa during and after cryopreservation: Structural and ultrastructural insights. Int J Vet Sci Med 2018;6:S49–S56. 30761321 10.1016/j.ijvsm.2017.11.001 PMC6161860

[gaad031-B24] Krieger M. Charting the fate of the “good cholesterol”: identification and characterization of the high-density lipoprotein receptor SR-BI. Annu Rev Biochem 1999;68:523–558. 10872459 10.1146/annurev.biochem.68.1.523

[gaad031-B25] Lane M , Thérien I, Moreau R, Manjunath P. Heparin and high-density lipoprotein mediate bovine sperm capacitation by different mechanisms. Biol Reprod 1999;60:169–175. 9858502 10.1095/biolreprod60.1.169

[gaad031-B26] Lee JA , Spidlen J, Boyce K, Cai J, Crosbie N, Dalphin M, Furlong J, Gasparetto M, Goldberg M, Goralczyk EM, et al; International Society for Advancement of Cytometry Data Standards Task Force. MIFlowCyt: the minimum information about a flow cytometry experiment. Cytometry Part A 2008;73:926–930. 10.1002/cyto.a.20623 PMC2773297 18752282

[gaad031-B27] Longobardi V , Albero G, De Canditiis C, Salzano A, Natale A, Balestrieri A, Neglia G, Campanile G, Gasparrini B. Cholesterol-loaded cyclodextrins prevent cryocapacitation damages in buffalo (*Bubalus bubalis*) cryopreserved sperm. Theriogenology 2017;89:359–364. 27793453 10.1016/j.theriogenology.2016.09.048

[gaad031-B28] Lu X , Liu J, Hou F, Liu Z, Cao X, Seo H, Gao B. Cholesterol induces pancreatic β cell apoptosis through oxidative stress pathway. Cell Stress Chaperones 2011;16:539–548. 21472505 10.1007/s12192-011-0265-7 PMC3156264

[gaad031-B29] Manjunath P , Thérien I. Role of seminal plasma phospholipid-binding proteins in sperm membrane lipid modification that occurs during capacitation. J Reprod Immunol 2002;53:109–119. 11730909 10.1016/s0165-0378(01)00098-5

[gaad031-B30] Marquez B , Suarez SS. Different signaling pathways in bovine sperm regulate capacitation and hyperactivation1. Biol Reprod 2004;70:1626–1633. 14766720 10.1095/biolreprod.103.026476

[gaad031-B31] Maxwell WM , Welch GR, Johnson LA. Viability and membrane integrity of spermatozoa after dilution and flow cytometric sorting in the presence or absence of seminal plasma. Reprod Fertil Dev 1996;8:1165–1178. 8981641 10.1071/rd9961165

[gaad031-B32] Medeiros CMO , Forell F, Oliveira ATD, Rodrigues JL. Current status of sperm cryopreservation: why isn’t it better? Theriogenology 2002;57:327–344. 11775978 10.1016/s0093-691x(01)00674-4

[gaad031-B33] Moore AI , Squires EL, Graham JK. Adding cholesterol to the stallion sperm plasma membrane improves cryosurvival. Cryobiology 2005;51:241–249. 16122725 10.1016/j.cryobiol.2005.07.004

[gaad031-B34] Moreno RD , Alvarado CP. The mammalian acrosome as a secretory lysosome: new and old evidence. Mol Reprod Dev 2006;73:1430–1434. 16894549 10.1002/mrd.20581

[gaad031-B35] Okazaki T , Abe S, Shimada M. Improved conception rates in sows inseminated with cryopreserved boar spermatozoa prepared with a more optimal combination of osmolality and glycerol in the freezing extender. Anim Sci J 2009;80:121–129. 20163581 10.1111/j.1740-0929.2008.00612.x

[gaad031-B36] Okazaki T , Akiyoshi T, Kan M, Mori M, Teshima H, Shimada M. Artificial insemination with seminal plasma improves the reproductive performance of frozen-thawed boar epididymal spermatozoa. J Androl 2012;33:990–998. 22282435 10.2164/jandrol.111.015115

[gaad031-B37] Okazaki T , Yoshida S, Teshima H, Shimada M. The addition of calcium ion chelator, EGTA to thawing solution improves fertilizing ability in frozen-thawed boar sperm. Anim Sci J 2011;82:412–419. 21615834 10.1111/j.1740-0929.2010.00856.x

[gaad031-B38] Purdy PH , Fox MH, Graham JK. The fluidity of Chinese hamster ovary cell and bull sperm membranes after cholesterol addition. Cryobiology 2005;51:102–112. 15993877 10.1016/j.cryobiol.2005.05.004

[gaad031-B39] Purdy PH , Graham JK. Effect of adding cholesterol to bull sperm membranes on sperm capacitation, the acrosome reaction, and fertility. Biol Reprod 2004;71:522–527. 15070825 10.1095/biolreprod.103.025577

[gaad031-B40] Quinn PJ , White IG. Phospholipid and cholesterol content of epididymal and ejaculated ram spermatozoa and seminal plasma in relation to cold shock. Aust J Biol Sci 1967;20:1205–1215. 6081218 10.1071/bi9671205

[gaad031-B41] Rajoriya JS , Prasad JK, Ramteke SS, Perumal P, Ghosh SK, Singh M, Pande M, Srivastava N. Enriching membrane cholesterol improves stability and cryosurvival of buffalo spermatozoa. Anim Reprod Sci 2016;164:72–81. 26619942 10.1016/j.anireprosci.2015.11.014

[gaad031-B42] Sieme H , Oldenhof H, Wolkers WF. Sperm membrane behaviour during cooling and cryopreservation. Reprod Domest Anim 2015;50 Suppl 3:20–26. 10.1111/rda.12594 26382025

[gaad031-B43] Srivastava N , Srivastava S, Ghosh S, Kumar A, Perumal P, Jerome A. Acrosome membrane integrity and cryocapacitation are related to cholesterol content of bull spermatozoa. Asian Pacific J Reprod 2013;2:126–131.

[gaad031-B44] Thérien I , Soubeyrand S, Manjunath P. Major proteins of bovine seminal plasma modulate sperm capacitation by high-density lipoprotein. Biol Reprod 1997;57:1080–1088. 9369174 10.1095/biolreprod57.5.1080

[gaad031-B45] Veatch SL , Keller SL. Organization in lipid membranes containing cholesterol. Phys Rev Lett 2002;89:268101. 12484857 10.1103/PhysRevLett.89.268101

[gaad031-B46] Vishwanath R , Shannon P. Storage of bovine semen in liquid and frozen state. Anim Reprod Sci 2000;62:23–53. 10924819 10.1016/s0378-4320(00)00153-6

[gaad031-B47] Watson PF , Morris GJ. Cold shock injury in animal cells. Symp Soc Exp Biol 1987;41:311–340. 3332489

[gaad031-B48] Witte TS , Schäfer-Somi S. Involvement of cholesterol, calcium and progesterone in the induction of capacitation and acrosome reaction of mammalian spermatozoa. Anim Reprod Sci 2007;102:181–193. 17870257 10.1016/j.anireprosci.2007.07.007

[gaad031-B49] Yadav HP , Kumar A, Shah N, Chauhan DS, Saxena A, Yadav S, Swain DK. Effect of cholesterol loaded cyclodextrin supplementation on tyrosine phosphorylation and apoptosis like changes in frozen thawed Hariana bull spermatozoa. Theriogenology 2017;96:164–171. 28532835 10.1016/j.theriogenology.2017.04.016

[gaad031-B50] Yang AJ , Chandswangbhuvana D, Margol L, Glabe CG. Loss of endosomal/lysosomal membrane impermeability is an early event in amyloid Abeta1-42 pathogenesis. J Neurosci Res 1998;52:691–698. 9669318 10.1002/(SICI)1097-4547(19980615)52:6<691::AID-JNR8>3.0.CO;2-3

[gaad031-B51] Zhu Z , Kawai T, Umehara T, Hoque SAM, Zeng W, Shimada M. Negative effects of ROS generated during linear sperm motility on gene expression and ATP generation in boar sperm mitochondria. Free Radic Biol Med 2019;141:159–171. 31212063 10.1016/j.freeradbiomed.2019.06.018

