# Supplementary material for: Treatment with cholesterol just after thawing maintains the fertility of bull sperm
Source: Mol Hum Reprod. 2023 Sep 1;29(9):gaad031. doi: 10.1093/molehr/gaad031 (PMC10502238; doi:10.1093/molehr/gaad031)
Supplement: gaad031_Supplementary_Data [file gaad031_supplementary_data.docx]

**Treatment with cholesterol just after thawing maintains the fertility of bull sperm**

Md. Mazharul Islam, Takashi Umehara, Natsumi Tsujita, Masanori Koyago, Masayuki Shimada

**Supplementary Information**

Supplementary Figure S1. Gating strategy of flow cytometry.

Supplementary Figure S2. Specificity of the antibody recognized SR-BI.

Supplementary Figure S3. Incorporation of BODIPY-cholesterol into fresh or frozen/thawed sperm.

Supplementary Figure S4. Concentration of cholesterol in the sperm after cholesterol treatment.

Supplementary Figure S5. Motility parameters after cholesterol treatment until 120 min.

Supplementary Figure S6. Straight velocity of fresh sperm after washing with cholesterol until 120 min.

Supplementary Figure S7. Pronuclear formation after incubation or fertilization.


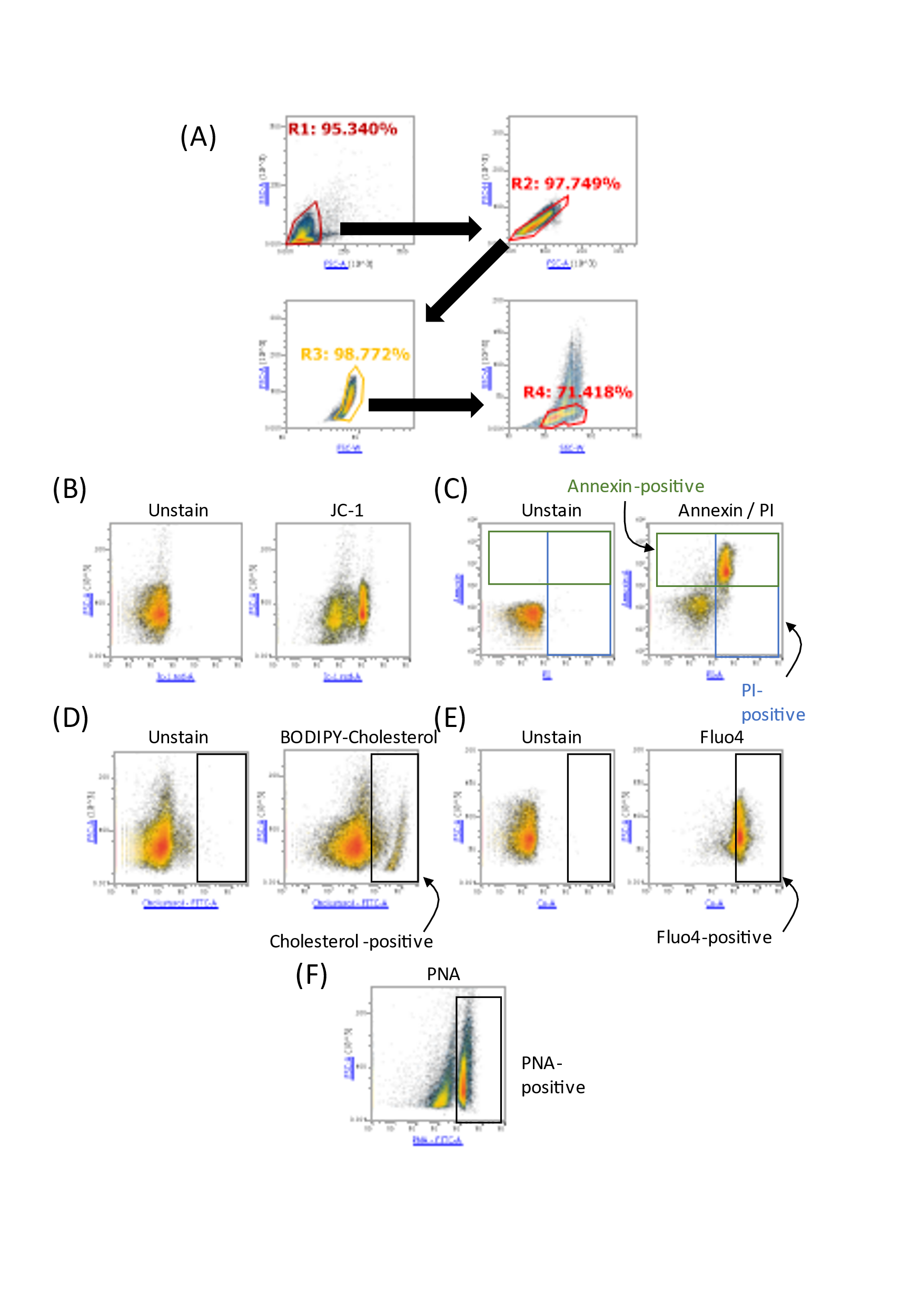


**Supplementary Figure S1. Gating strategy of flow cytometry.**

1. Gating strategy for the selection of single cell. Using forward scatter (FSC)-A and side scatter (SSC)-A dot plots, similar size and complexity cells were firstly selected (R1). In FSC-A and FSC-H dot plots and FSC-A and FSC-W dot plots, similar-size cells were accumulated near area; thus, using these plots, again similar-size cells were selected (R2, R3). Finally, similar complexity cells were again selected using SSC-A and SSC-W (R4). The cells in R4 were used for below analysis.
2. Dot plots of FSC-A and 5,5’,6,6’-tetrachloro-1,1’,3,3’-tetraethylbenzimidazolyl carbocyanine iodide (JC-1) red. Average of fluorescence intensity (MFI) of JC-1 red was used for the analysis.
3. Dot plots of propidium iodide (PI) and Annexin-V. The cells in the blue box were recognized as PI-positive cells, and the cells in the green box were recognized as Annexin V-positive cells.
4. Dot plots of FSC-A and boron-dipyrromethene (BODIPY)-cholesterol. The cells in the black box were recognized as cholesterol-incorporated cells.
5. Dot plots of FSC-A and Fluo-4. The cells in the black box were recognized as Fluo4-positive cells.
6. Dot plots of FSC-A and peanut agglutinin lectin (PNA). The cells in the black box were recognized as PNA-positive cells.


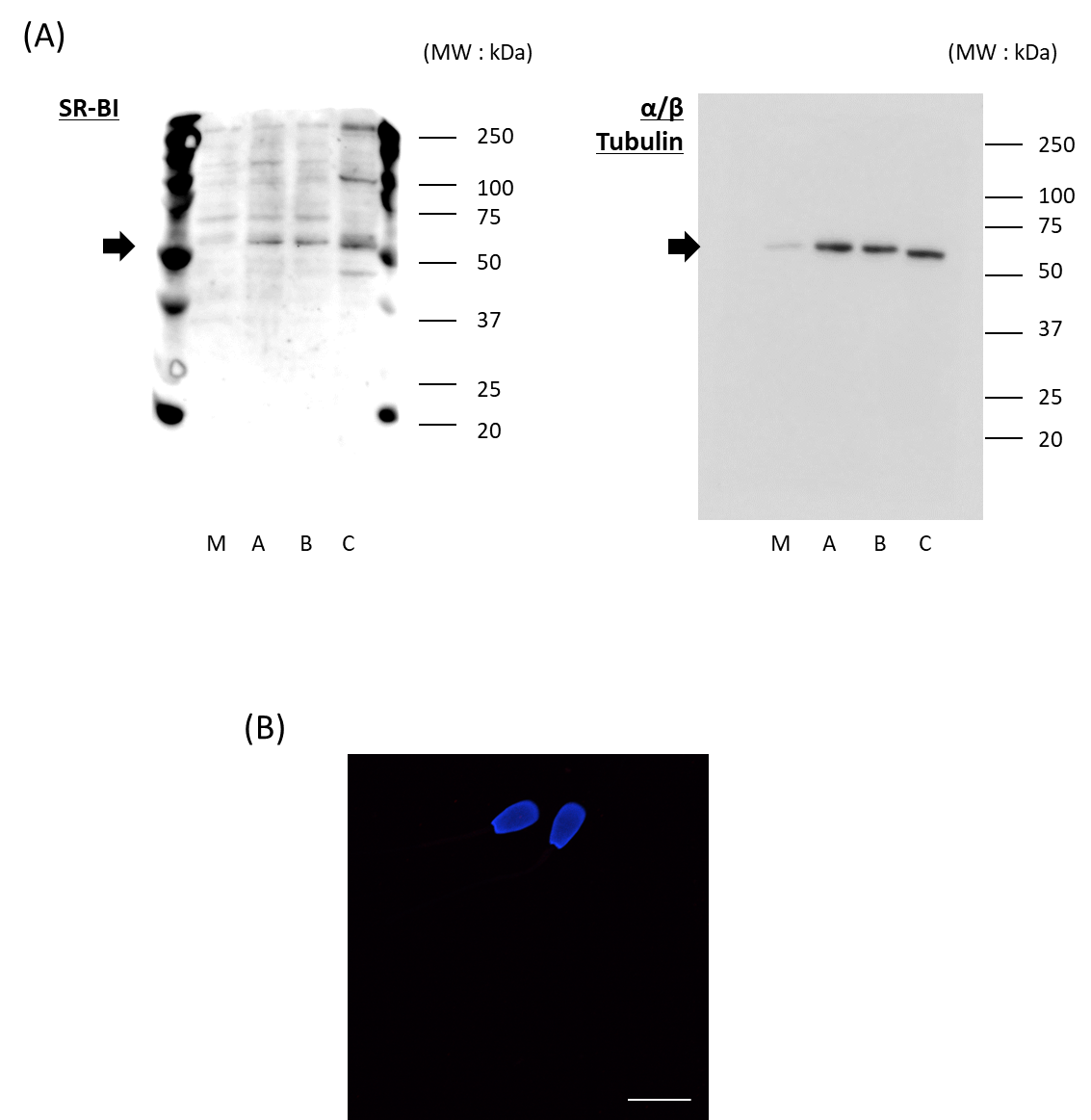


**Supplementary Figure S2. Specificity of the antibody recognized SR-BI.**

1. Western blotting using anti scavenger receptor class B Type I (SR-BI) antibody and anti-a/b tubulin antibody. Black arrows indicated the expected bands. M : mouse sperm A~C: each bull sperm
2. Fluorescence image after staining only secondary antibody without primary antibody. DAPI was used as counter staining.


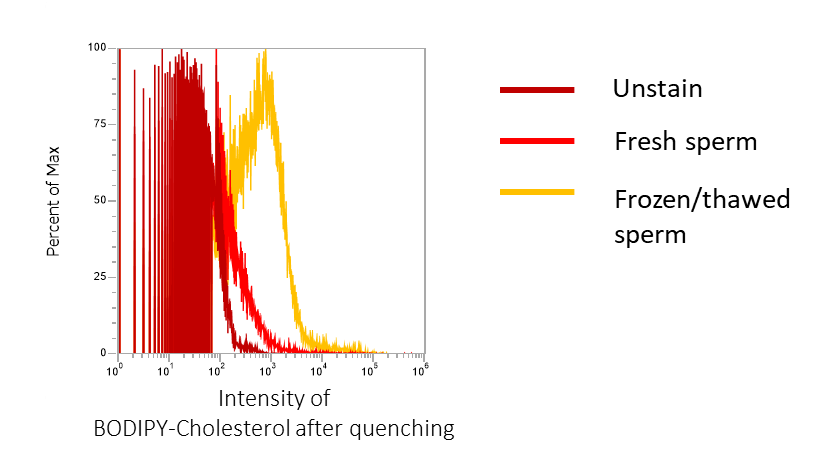


**Supplementary Figure S3. Incorporation of boron-dipyrromethene (BODIPY)-cholesterol into fresh or frozen/thawed sperm.**

**Supplementary Figure S4. Concentration of cholesterol in the sperm after cholesterol treatment.**

CTRL: washing without cholesterol

CHL: washing with cholesterol

CHL+BLT-1: washing with cholesterol plus block lipid transport 1 (BLT-1)


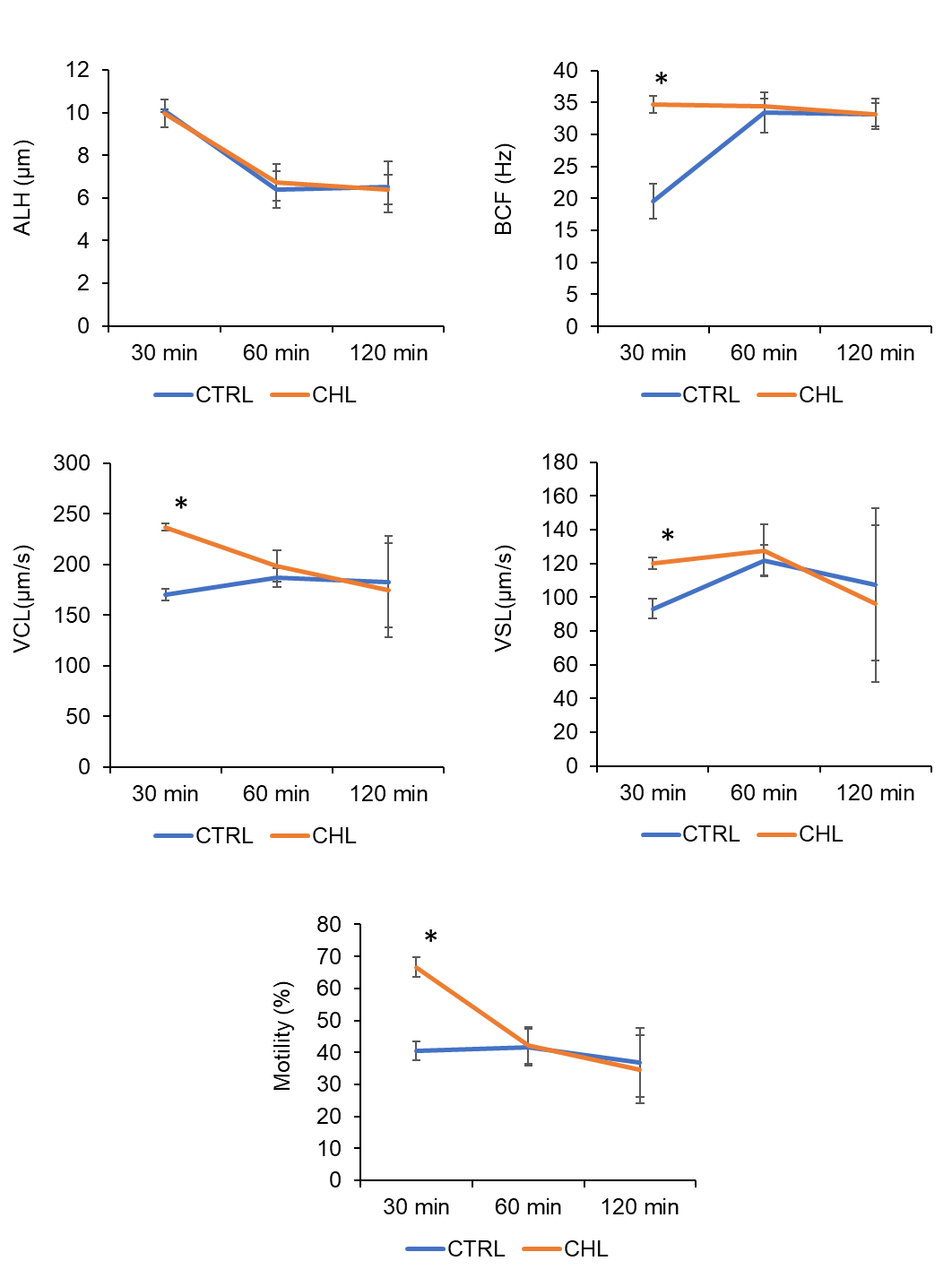


**Supplementary Figure S5. Motility parameters after cholesterol treatment until 120 min.**

* indicates the significantly differences (P<0.05) compared with control (CTRL).

CHL: washing with cholesterol ALH : amplitude lateral head displacement BCF : beat cross frequency VCL : curvilinear velocity VSL : straight line velocity


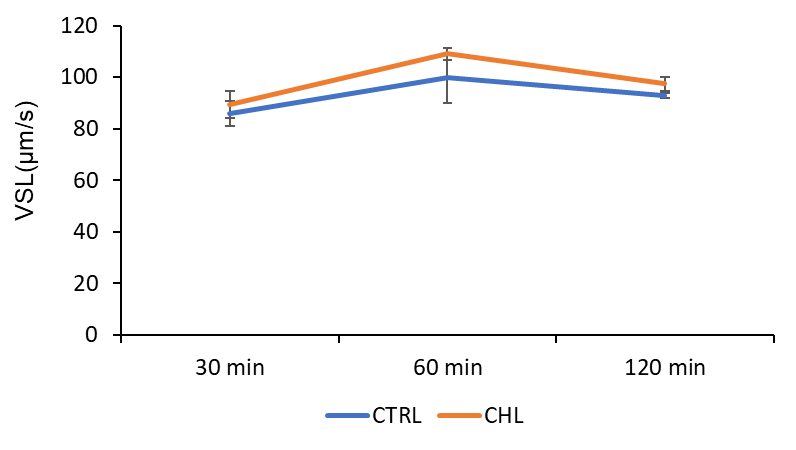


**Supplementary Figure S6. Straight velocity (VSL) of fresh sperm after washing with cholesterol until 120 min.**

CTRL: washing without cholesterol

CHL: washing with cholesterol


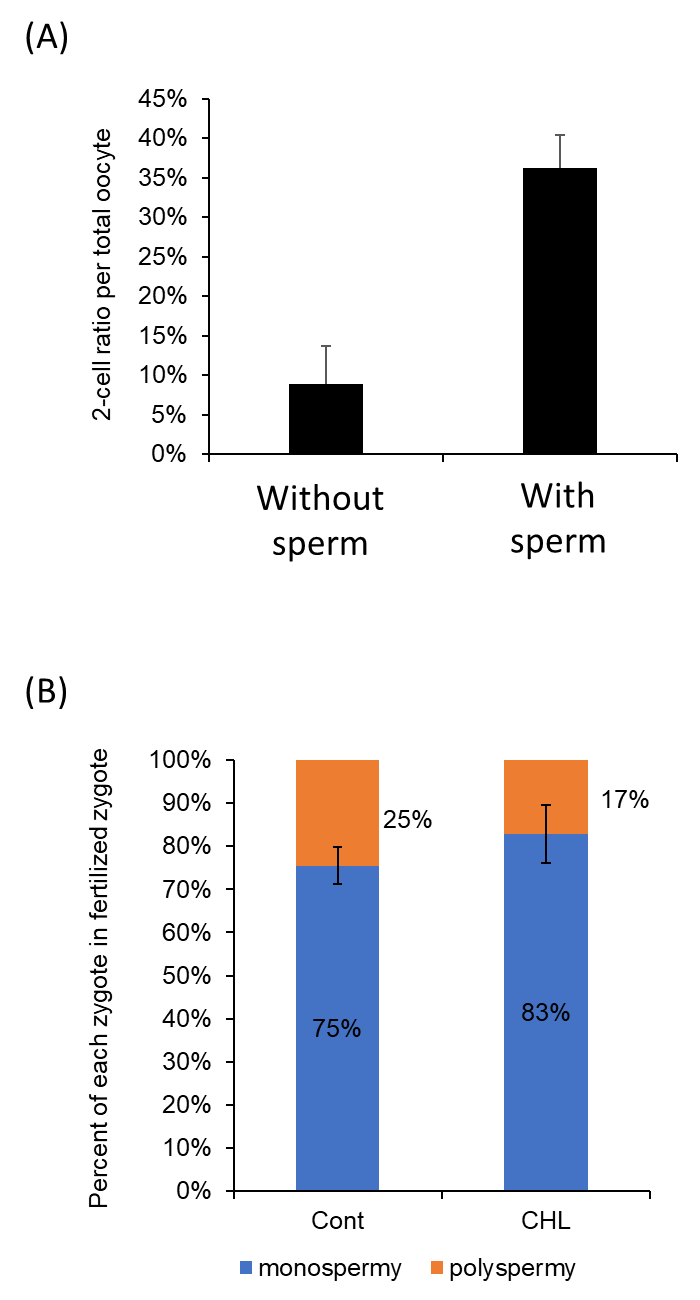


**Supplementary Figure S7. Pronuclear formation after incubation or fertilization.**

1. 2-cell ratio after 48 hr culture with/without sperm
2. Number of pronuclear after 18 hr from insemination

Cont: washing without cholesterol, CHL: washing with cholesterol
